# The Dark Side of High-Fliers: The Dark Triad, High-Flier Traits, Engagement, and Subjective Success

**DOI:** 10.3389/fpsyg.2021.647676

**Published:** 2021-04-27

**Authors:** Adrian Furnham, Luke Treglown

**Affiliations:** ^1^BI: Norwegian Business School, Nydalsveien, Oslo, Norway; ^2^Thomas International, Marlow, United Kingdom

**Keywords:** personality, dark triad, engagement, work success, high potential trait indicator, mediation analysis, SEM

## Abstract

The aim of this study was to understand the relationship between bright-side, High Potential and dark-side Dark Triad traits, as well as work engagement on judgements of perceived success. In all, 290 working adults completed questionnaires assessing their High Potential Personality Traits (HPTI), their dark-triad traits, job engagement and self-rated success at work. The data showed that the three dark-triad traits (Narcissism, Psychopathy, Machiavellianism) were systematically and significantly correlated with High Potential traits Adjustment/neuroticism, Tolerance of Ambiguity and Conscientiousness. Three HPTI traits, namely curiosity, Conscientiousness, and courage, were systematically positively correlated with all three engagement measures. Narcissism was strongly related to all measures of engagement. Those with higher scores Adjustment, Courage, and Narcissism and of the male sex, rated their success highest. Job engagement mediated between high-flier and dark-side traits and success ratings. Implications and limitations are discussed.

## Introduction

This study attempts to understand the relationship between high-flier traits, the dark triad traits, job engagement and subjectively defined success. It is part of a systemic research programme on leadership derailment (Gøtzsche-Astrup et al., 2016; Treglown et al., 2016; Teodorescu et al., 2017). The primary aim of the study is to examine how “bright-side” traits, measured by new and validated measure of high-flying traits (HPTI), and a measure of “dark-side” traits measured by the well-established Dirty Dozen measure (Jonason and Webster, 2010) correlate with Work Engagement and subjectively related success. It extends the work of MacRae and Furnham (in press) and Teodorescu et al. (2017) by first using the Dark Triad (DT) measure, but also looking at work engagement as an outcome measure.

There is a diverse, but growing, literature on the relationship between bright-side personality traits and dark-side traits and disorders (Furnham, 2021). There are two issues of interest: the relationship between these two different concepts and measures; and the extent to which they correlate with success and failure at work. This study looks at a high-flier trait measure of the bright-side, and the DT measure of the dark-side, and how they are inter-related and correlate with job engagement and subjective success. Our major interest was on job success and the issue of incremental validity of the DT and work engagement over bright-side traits in predicting work success. We also explored the idea that job engagement mediated the relationship between bright- and dark-side personality and job success. Some studies have suggested that various factors moderate the relationship between personality and job outcome variables (Furnham, 2018) but believe certain factors are just as likely to mediate this relationship, as they are often directly related to the job like organizational size and job tenure (Spurk et al., 2016).

There is a growing literature on dark-side and specifically DT traits and job success and failure. Dysfunctional personality traits impair an employee's efforts to “get along” with co-workers which will handicap their efforts to “get ahead” of others (Hogan, 2009). Because those personalities that comprise the DT are so manipulative, selfish, and self-serving, their evaluation of costs, benefits, obligations, and reciprocity as well as their callousness undermines work relationships (Furnham et al., 2014). LeBreton et al. (2018) noted that research on the link between DT traits and job performance has been inconclusive. Lots of work on Machiavellianism, less on Narcissism and until recently little on Psychopathy. The mixed evidence results partly from the use of different measures used, O'Boyle et al. (2012), in a major meta-analysis looked at DT traits, job performance and counterproductive workplace behaviors. They found that both Machiavellianism and Psychopathy have significant predicted relationships with job performance, but that Narcissism does not. They argued that a simple bivariate relationship between DT traits and job performance may be an oversimplification and researchers should consider possible moderators of the relationship between the DT and job performance.

In a very relevant paper to this Spurk et al. (2016) examined DT correlates of both objective and subjective measures of success in nearly 800 German managers. They found that after controlling for other relevant variables (such as gender, age, job tenure, organization size, etc.), narcissism was positively related to salary, and Machiavellianism positively related to leadership position and career satisfaction, but Psychopathy was negatively related to all of their measures. They noted “Hence, whether bad guys get ahead or fall behind seems to depend on the type of dark trait.” (p117). In this study we examine both bright- and dark-side traits.

### High-Flier Traits

MacRae and Furnham (2014), Furnham et al. (2020) designed and validated a reliable and comprehensive measure of personality at work (HPTI). It aimed to identify High-Fliers by examining those characteristics linked to long term work success, but which are not dependent on experience or knowledge. The HPTI factors used to assess potential at work are Conscientiousness (which is a FFM personality trait that is characterized by self-discipline, organization, and ability to moderate one's own impulses), Adjustment (which is the adaptive end of the FFM Neuroticism trait characterized by recurrent emotional resilience to stressors, positive affect and perceptions), and Curiosity (this like Openness is a Big Five personality trait characterized by curiosity for new ideas, experiences, and situations). Ambiguity Tolerance (also known as Ambiguity Tolerance describes how an individual processes and perceives unfamiliarity or incongruence), Courage (is the ability to combat or mitigate negative or threat-based emotions and broaden the potential range of responses, and is exhibited as the willingness to confront difficult situations and solve problems in spite of adversity), and Competitiveness (which is a distinct trait related to need for achievement and A type behavior and foceses on the drive self-improvement, individual and team success, and learning).

The HPTI has received careful validation and been used in several published studies (Furnham and MacRae, 2020; Treglown et al., 2020a,b). In a psychometric evaluation of this study Furnham and MacRae (2020) demonstrated that the internal reliability of each trait was satisfactory, as was the structure of the inventory, as assessed by confirmatory factor analysis. Females scored higher on Conscientiousness and Curiosity but lower on Courage compared to males. The HPTI showed concurrent validity with the Big Five as well as measures of engagement. The six factors accounted for 30% of the variance in predicting self-rated work success.

MacRae (2012) established the factor structure of the HPTI using confirmatory factor analysis as well as the construct validity of the scale by showing how the traits were significantly related to subjective and objective measures of career success. Teodorescu et al. (2017) found the scale predictably related to a person's history of promotion. Using a variety of outcome measures like income, promotional speed and subjective ratings, they showed age, and gender, but also Conscientiousness, Competitiveness, Adjustment, and Ambiguity Acceptance, could account for between 10 and 20% of the variance with respect to success at work. Other studies in different settings has demonstrated its predictive and incremental validity (Martinsen et al., in press).

### Dark Triads Traits

There is a relatively new area of research into a concept called the “Dark Triad” which is an individual differences construct proposed by Paulhus and Williams (2002). The use of the term “dark” reflects the idea that these traits, in effect sub-clinical personality disorders, independently and together have interpersonally aversive qualities. They are Narcissism which characterized by a vanity, arrogance egotism and a lack of empathy; Machiavellianism is manifest by manipulative exploitation of others, a cynical disregard for laws and morals and a focus on self-interest and deception, and psychopathy is characterized by antisocial behavior, impulsivity, selfishness, callousness, and remorselessness. Those with high Dark Triad scores seem totally insensitive to the feelings of others and deeply cynical about human nature.

When Paulhus and Williams (2002) introduced the Dark Triad they did so because they noticed an overlap of similar features, specifically “all three entail a socially malevolent character with behavior tendencies toward self-promotion, emotional coldness, duplicity, and aggressiveness” (p. 557). There are now many hundreds of papers using the dark triad and various reviews of the literature (Furnham et al., 2013; Andersen et al., in press).

Jonason et al. (2010) proposed three explanations for how the Dark Triad traits would be functionally adaptive. They first proposed that because those who score higher on the Dark Triad are conceptualized as more *agentic*, meaning proactive in the manipulation of their environments, they are more successful in the control of outcomes, and more accurate in their perceptions. The second proposition is that narcissism facilitates *social exploitation* through high degrees of self-confidence because communicating confidently increases persuasiveness. The *third* proposition is related to those who score higher on the Dark Triad and their exploitation of high-risk, high-payoff niches like “gambling for high stakes.”

Those who score high on the Dark-Triad are selfish and self-serving in their valuation of rewards and costs, willingness to overlook obligations and reciprocity, and lack of emotional commitment to others they are likely undermine work relationships. Machiavellians are cynical and distrustful, and less likely to assume that they will be paid reciprocally for any extra effort they put in on the job. Narcissists feel they always outperform their fellow co-workers so that rules about reciprocity and obligation do not apply to them. Psychopaths' celebrated insensitivity to others' means they are less likely to act in ways that will please or help others (Furnham et al., 2013).

In a meta-analysis of over 40,000 people O'Boyle et al. (2011) were able to show that the Dark Triad was associated with Counter-Productive Work Behavior. However, they found very little relationship between the Dark Triad and work performance because of the power of two moderating factors. The first was authority and showed the more power a DT person had because they were in higher positions of authority, the more dangerous they were and likely to cause problems. The second was organizational culture which showed the more the corporate culture was collectivistic and stressed organizational commitment and citizenship behavior the less the influence of those with DT. They concluded, as have others, that although the three DT traits are positively related, they are sufficiently distinctive enough “*to warrant theoretical and empirical partitioning”* (p. 557).

Others have argued that they are quite distinct in their functioning and measurement and need to be considered separately (Furnham et al., 2014). In this study we are interested in how they relate of engagement and subjectively rated success.

### Engagement and Disengagement

The concept of work engagement has emerged from research showing that certain employees find pleasure in work despite strenuous job requirements. This led Schaufeli et al. (2002) to propose the theoretical construct of work engagement, a fulfilling and positive work mindset. The scale is perhaps the most widely used in the area because of its facet structure and psychometric properties.

Work engagement is not momentary, but is a persistent and continuous affective and cognitive state, comprised of three dimensions*: absorption* (i.e., being focused and engrossed in work), *dedication* (i.e., being pride and enjoying work), and *vigor* (i.e., being energetic and resilience). There are many studies which have looked a personality trait correlates of work engagement (e.g., Rigg, 2013; Schaufeli, 2013; Salleh and Memon, 2015). They have tended to show that significant positive correlations particularly for Conscientiousness and Openness but negative correlation for Neuroticism. Equally, there appears to be very few studies on the relationship between engagement and the dark triad which this study addresses. An exception is the work of Filipkowski and Derbis (2020) who found that work engagement weakened the influence of the Dark Triad on counterproductive behaviors at work.

### Success at Work

Career success has been defined as “the accumulated positive work and psychological outcomes resulting from one's work experiences” (Seibert et al., 2001). In this study we are using a simple five item measure used by Teodorescu et al. (2017).

It has been suggested that one must distinguish between objective and subjective career success (Furnham, 2018). Objective success refers to extrinsic indicators of success, while subjective, or intrinsic, measures of career success attempt to capture an individual's subjective judgments about their career achievements and typically include self-report measures such as job or career satisfaction (Judge et al., 1999; Barrick et al., 2001).

Inevitably most researchers would prefer objective measures of success supplied by an organization from their files as this hopefully eliminates some of the biases. Yet whilst they might demonstrate important differences within organizations it may be very problematic to combine individual from many organizations without in some way weighting the data. Similarly, it is possible that different measures of “objective success” like job title or pay grade may be interpreted and weighted by people very differently.

Various studies used a mix of measures. Thus, Sutin et al. (2009) used occupational prestige (job title), annual income, and self-reported job satisfaction. Some studies using both objective (salary) and subjective (career) satisfaction found they had different antecedents (Hirschi and Jaensch, 2015). Abele and Spurk (2009) examined how objective and subjective measures of career success interrelate over time. They found that measures of subjective success had a strong influence on the growth of objective success. They concluded that subjective ratings influenced objective ratings more than vice versa, suggesting the power of self-confidence in career success. This suggests that in all research in this area it is best, if possible, to have multiple measures of both objective and subjective success if one really wants to understand the problem.

Three of the Big Five personality traits have been consistently linked to career success, namely Conscientiousness, Neuroticism (low Adjustment) and Openness to Experience (Curiosity) (Judge et al., 1999; Ng et al., 2005). A meta-analysis of the Big five personality traits and career success found Conscientiousness to be the strongest and most consistent predictor of career success across occupations and all measures of success (Barrick et al., 2001). Neuroticism (Low adjustment) has been found to negatively relate to job performance, as low reactivity to stress and anxiety may reduce both career satisfaction and effective career management, leading to poor performance (Judge et al., 1999; Seibert et al., 2001; Ng et al., 2005). Barrick et al. (2001) found that Openness to Experience (Curiosity) was less associated to job performance than Conscientiousness or Neuroticism. However, Curiosity may still be useful for identifying potential if made more relevant to the workplace such as openness to new ideas and approaches instead of aesthetic appreciation and emotionality.

Ng et al. (2005) argue objective and subjective measures of success may be conceptually distinct as evidenced by the weak correlations between each other and proposed they may be predicted by different factors. Ng et al. (2005) suggest personality traits may be more relevant for predicting subjective measures of success which are more strongly associated to psychological well-being and personal assessment, while human capital and demographics may be better predictors of objective measures of success. Teodorescu et al. (2017) found HPI traits Conscientiousness, Competitiveness, Adjustment, and Ambiguity Acceptance were significant predictors of subjective success accounting for 15.8% of the variation.

O'Boyle et al. (2012) and LeBreton et al. (2018) argued that there was a need to test beyond the direct effects of dark triad on job performance. Previous research models dark side personality to have a direct, derailing impact on organizational outcomes, independent of the context within which it occurs (Moscoso and Salgado, 2004). This paper aims to explore whether workplace engagement represents a mediating factor in the relationship between dark personality and job performance. There are literatures both on the role of personality traits in predicting which employees will be engaged at work (e.g., Barreiro and Treglown, 2020) as well as how engagement predicts increased job performance (e.g., Demerouti et al., 2010). However, two gaps exist: firstly, to what extent do dark personality traits hinder or help an employee to be engaged within the workplace?; secondly, to what extent does engagement mediate the relationship between individual differences and job performance? Exploring these questions will offer important insight into understanding how and why dark personality traits hinder employee job performance.

### This Study

We are concerned in this study with correlates of our subjective work success measure. First, we predicted that age and sex would be related to subjective success: age would correlate positively with success rating (H1); males, with their usual tendency to hubris, would give higher ratings than females (H2). Next both the total engagement score (H3) and all three facets (H4) would be correlated with the subjective success measure. Next, Narcissism (H5) would be correlated with subjective success. Of the HPTI traits, the following would be positively associated with subjective success ratings: Curiosity (H6), Conscientiousness (H7) Courage (H8) and Competitive (H9), and that low Adjustment (Neuroticism; H10) would be negative associated with Subject success. We were also interested in Job Engagement at the domain and facet level and hypothesized that Neuroticism would be negatively (H11) but Conscientiousness (H12) and Narcissism (H13) positively associated with Engagement. We also explored the idea that Engagement mediated the relationship between the bright- and dark-side traits and perceived success. In this study we explored the relationship between Machiavellianism and Psychopathy and both Engagement and Success but did not formulate any particular hypotheses, though from previous studies, notably Spurk et al. (2016) it may be expected that Machiavellianism is positively and Psychopathy negatively related to subjective job success.

## Method

### Participants

In all, 303 respondents originally participated in the study. However, due to incomplete responses, 16 individuals were removed. The final sample consisted of 181 males and 109 females. The mean age for the sample was 34.1 years (SD = 9.6). Most had completed their schooling and around 40% were graduates. Around a 1/2 worked in the public sector, 1/2 in the private sector, though it was not always possible to classify them accordingly.

### Materials

*The High Potential Traits Inventory* (HPTI) is a measure of normal, “bright” personality traits, designed to ascertain how individuals think, prioritize, and act in the workplace (MacRae and Furnham, 2014). The questionnaire comprises of 78 items, which participants decide the extent to which they agree upon a 7-point Likert scale (1 – Completely Disagree; 7 – Completely Agree). Previous research demonstrates that the HPTI assesses six dimensions of personality (MacRae, 2012): Conscientiousness, Adjustment, Curiosity, Risk Approach, Ambiguity, and Competitiveness.

*The Dirty Dozen* (Jonason and Webster, 2010) was used to measure the Dark Triad (DT) of Narcissism, Psychopathy, and Machiavellianism in a 12-item scale. Participants rate the extent to which they agree with the items on a five-point Likert scale (1 – Not Like Me At All; 5 – Very Much Like Me). The scale has reported high internal validity (Maples et al., 2014), ranging from α = 0.86 (Machiavellianism) to α = 0.90 (Psychopathy).

*Utrecht Work Engagement Scale (UWES)* (Schaufeli et al., 2002). The UWES is a 17-item inventory, where respondents indicate the extent to which they agree with the statements upon a 7-point Likert scale (0 = Never; 6 = Always). The questions are grouped into three broad subscales that identify different underlying facets of engagement: Vigor (6 items), Dedication (5 items), and Absorption (6 items). The three subscales have reported to have acceptable internal validity: Vigor α = 0.80; Dedication α = 0.91; Absorption α = 0.75 (Schaufeli et al., 2002).

*Work Success:* Participants were asked to respond to this 5-item questionnaire, rating their own perceived success at work. Participants rated their subjective success in five areas: General Success - “I am generally very successful”; Success with Promotions - “I do not get promoted as quickly as my colleagues”; Success in Education - “I was/am very successful in education”; Success with Marks - “In education, I tend(ed) to receive higher marks than my peers”; Success at Work- “I am very successful in my line of work.” Participants would decide the extent of their agreement to that statement on a 7-point Likert scale (1 = Strongly Disagree; 7 = Strongly Agree). The mean score for the sum of the five items was 24.62 (SD = 5.13) and the five-item scale had a Cronbach's alpha of 0.72, similar to what has been seen in previous studies using this scale (Teodorescu et al., 2017).

### Procedure

Participants were recruited through Amazon Mechanical Turk (MTurk), an online market for recruiting workers to participate in research and surveys. Researchers have found that MTurk yields data that is at least as reliable as other traditional recruitment methodologies, benefiting from marginally greater diversity than standard Internet surveys (Buhrmester et al., 2016). The six questionnaires were hosted on MTurk, for which workers were paid $3 for successfully completing the questionnaires.

## Results

### Descriptive Statistics

Table 1 shows the descriptive statistics and sex differences and Table 2 the alphas of the subscales for each questionnaire. All but one subscale (*Ambiguity* within the HPTI) have alphas that exceed 0.70, indicating sufficient internal reliability (Yang and Green, 2011). Sex differences were found with the HPTI Conscientiousness subscale, with women scoring significantly higher (*p* < *0.0*1) compared to men. No sex differences were noted in the Dirty Dozen dimensions. Women scored significantly higher on Work Engagement dimensions vigor (*p* = 0.014) and dedication (*p* = 0.048).

**Table 1:** Sex differences in the HPTI, Dirty Dozen, UWES, and Subjective Success subscales.

**Scale**	**Items**	**Male** ***(N = 181)***		**Female** ***(N = 109)***		***F* statistic** **(1,289)**
		**Mean**	**SD**	**Mean**	**SD**	
*HPTI*	*78*					
Neuroticism	13	49.7	16.4	45.7	18.4	3.58
Curiosity	13	65.0	11.1	64.8	10.7	0.04
Ambition	13	45.5	10.2	46.4	10.0	0.44
Conscientiousness	13	62.1	11.2	66.2	10.6	9.59^**^
Courage	13	54.2	12.8	52.4	13.1	1.32
Competitiveness	13	57.1	9.95	57.5	9.78	0.103
*Dirty Dozen*	*12*					
Narcissism	4	17.0	6.62	16.2	7.06	0.89
Psychopathy	4	14.2	6.61	12.7	7.14	3.19
Machiavellianism	4	13.8	6.98	12.9	7.90	0.87
*UWES Work Engagement*	*17*					
Vigor	6	4.87	1.26	5.23	1.08	6.15^*^
Dedication	5	4.93	1.46	5.27	1.27	3.94^*^
Absorption	6	4.71	1.35	5.01	1.25	3.56
*Subjective Success*	5	24.8	5.38	26.7	4.78	8.63^**^

* *= p < 0.05;*

** *= p < 0.001*.

**Table 2:** Correlations between HPTI, Dirty Dozen, UWES subscales, and Subjective Success.

	**M (SD)**	**α**	**1**	**2**	**3**	**4**	**5**	**6**	**7**	**8**	**9**	**10**	**11**	**12**	**13**
1. Neuroticism	47.7 (16.7)	0.86													
2. Curiosity	64.7 (10.8)	0.80	−0.05												
3. Ambiguity	46.0 (10.0)	0.61	−0.55^**^	0.02											
4. Conscient.	63.7 (11.2)	0.78	−0.52^**^	0.46^**^	0.13^**^										
5. Courage	57.5 (10.0)	0.70	−0.38^**^	0.50^**^	0.29^**^	0.64^**^									
6. Competitive.	53.5 (12.9)	0.74	0.10^**^	0.32^**^	−0.08	0.33^**^	0.47^**^								
7. Narcissism	16.5 (6.76)	0.89	0.54^**^	0.11	−0.31^**^	−0.23^**^	−0.10	0.14^*^							
8. Psychopathy	13.4 (6.76)	0.91	0.75^**^	0.04	−0.38^**^	−0.41^**^	−0.21^**^	0.10	0.64^**^						
9. Mach.	13.3 (7.27)	0.94	0.67^**^	0.11	−0.30^**^	−0.33^**^	−0.14	0.17^*^	0.63^**^	0.82^**^					
10. Vigor	29.9 (7.14)	0.86	−0.05	0.23^**^	−0.07	0.27^**^	0.30^**^	0.13	0.34^**^	0.11	0.13				
11. Dedication	25.2 (6.97)	0.92	−0.01	0.22^**^	−0.09	0.24^**^	0.23^**^	0.15^*^	0.40^**^	0.08	0.12	0.88^**^			
12. Absorption	28.7 (7.83)	0.89	0.14^*^	0.24^**^	−0.17^*^	0.16^**^	0.19^**^	0.18^**^	0.43^**^	0.20^**^	0.21^**^	0.84^**^	0.88^**^		
13. Subjective Success	25.5 (5.25)	0.72	−0.16^*^	0.23^**^	−0.01	0.30^**^	0.30^**^	0.12	0.26^**^	0.02	0.01	0.69^**^	0.67^**^	0.62^**^	

* *= p < 0.05;*

** *= p < 0.001*.

### HPTI, Dark Triad, Work Engagement, and Subjective Success

Table 2 shows that subjective success was significantly positively correlated with three HPTI traits (Courage, Conscientiousness, and Ambiguity), Narcissism, and very highly (*r* > 0.60) all three UWES scales. Curiosity was the only negative correlate of Subjective Success. Thus, all of the hypotheses except H9 was confirmed.

Also higher Neuroticism and lower Conscientiousness were significantly negatively correlated with the dark triad. Additionally, Ambiguity was negatively correlated with Narcissism and Psychopathy, Courage correlated negatively with Psychopathy, and Competitiveness correlated positively with Narcissism and Machiavellianism. Curiosity, Conscientiousness, Courage, and Competitiveness were significantly positively correlated with the three UWES subscales. Absorption was positively correlated with Machiavellianism and Psychopathy, whilst Narcissism was positively correlated with all three subscales.

Three two-step hierarchical regressions were conducted to analyse which HPTI and Dark Triad traits were predictive of the three Work Engagement subscales (results can be seen in Tables 3–5). The first contained demographic variables (age and gender), which was only significant for Vigor (Gender; *ß* = −0.12, *p* = 0.043). The second step included all six HPTI subscales and the three Dark Triad traits. Higher levels of Courage and Psychopathy, whilst lower levels of Neuroticism and Ambiguity, were predictive of Vigor, explaining 28.9% of the variance. Higher Narcissism was the only significant predictor for Dedication (30.1% of variance explained) and Absorption (26.3% of variance explained).

**Table 3:** Regression of HPTI and Dark Triad on UWES Dedication.

	**Engagement - Dedication**	
	***ß***	***t***
**Step 1**		
Age	0.02	0.31
Gender	−0.10	−1.57
*Adj.R^2^*		0.00
*F Change*		*F*(282) = 1.46
**Step 2**		
HPTI – Neuroticism	−0.14	−1.47
HPTI – Curiosity	0.02	0.38
HPTI – Ambiguity	−0.09	−1.43
HPTI – Conscientiousness	0.14	1.70
HPTI – Competitiveness	−0.03	−0.44
HPTI – Courage	0.15	1.85
Narcissism	0.55	8.37^***^
Psychopathy	−0.11	−1.10
Machiavellianism	−0.01	−0.13
*R^2^*		0.30
*ΔR^2^*		0.29
*F Change*		*F*(273) = 12.6^***^

*** *p < 0.001*.

**Table 4:** Regression of HPTI and Dark Triad on UWES Vigor.

	**Engagement - Vigor**	
	***ß***	***t***
**Step 1**		
Age	0.01	0.20
Gender	−0.12	−2.03^*^
*Adj.R^2^*		0.02
*F Change*		*F*(282) = 2.27
**Step 2**		
HPTI – Neuroticism	−0.24	−2.50^*^
HPTI – Curiosity	−0.01	−0.17
HPTI – Ambiguity	−0.14	−2.04^*^
HPTI – Conscientiousness	0.14	1.62
HPTI – Competitiveness	−0.10	−1.48
HPTI – Courage	0.27	3.40^**^
Narcissism	0.43	6.29^***^
Psychopathy	0.04	0.37
Machiavellianism	0.05	0.50
*R^2^*		0.29
*ΔR^2^*		0.27
*F Change*		*F*(273) = 11.6^***^

* *p < 0.05;*

** *p < 0.01;*

*** *p < 0.001*.

**Table 5:** Regression of HPTI and Dark Triad on UWES Absorption.

	**Engagement - Absorption**	
	***ß***	***t***
**Step 1**		
Age	0.01	0.20
Gender	−0.09	−1.45
*Adj.R^2^*		0.01
*F Change*		*F*(283) = 1.18
**Step 2**		
HPTI – Neuroticism	−0.02	−0.22
HPTI – Curiosity	0.07	1.14
HPTI – Ambiguity	−0.11	−1.58
HPTI – Conscientiousness	0.11	1.26
HPTI – Competitiveness	−0.02	−0.27
HPTI – Courage	0.15	1.86
Narcissism	0.47	6.70^***^
Psychopathy	−0.03	−0.28
Machiavellianism	−0.03	−0.30
*R^2^*		0.26
*ΔR^2^*		0.25
*F Change*		*F*(272) = 10.5^***^

*** *p < 0.001*.

A three-step hierarchical regression was conducted to assess how the HPTI traits, Dark Triad, and engagement factors predicted Subjective Success (results in Table 6). The first step contained demographic variables (age and gender), which was only significant for Vigor (Gender; *ß* = −0.15, *p* = 0.013). The second step included the six HPTI subscales and three Dark Triad traits. This step explained an additional 23.8% of the variance, with Neuroticism negatively, and Courage and Narcissism positively predicting Subjective Success. The third step entered the three UWES subscales, explaining an additional 25.9% of the variance (52.8% total). In this final step, the only significant predictors were Vigor and Dedication, both of which positively predicted Subjective Success.

**Table 6:** Regression of HPTI, Dark Triad, and UWES on Subjective Success.

	**Subjective Success**	
	***ß***	***t***
**Step 1**		
Age	0.06	1.04
Gender	−0.15	−2.51^*^
*ΔR^2^*		0.03
*F Score*		*F*(282) = 4.44^*^
**Step 2**		
HPTI – Neuroticism	−0.34	−3.40^**^
HPTI – Curiosity	0.04	0.59
HPTI – Ambiguity	−0.11	−1.71
HPTI – Conscientiousness	0.09	1.11
HPTI – Competitiveness	−0.04	−0.54
HPTI – Courage	0.19	2.36^*^
Narcissism	0.41	5.90^***^
Psychopathy	0.13	1.22
Machiavellianism	−0.07	−0.68
*ΔR^2^*		0.23
*F Score*		*F*(272) = 9.87^***^
**Step 3**		
Vigor	0.29	2.99^**^
Dedication	0.27	2.49^*^
Absorption	0.08	0.85
*ΔR^2^*		0.26
*F Score*		*F*(270) = 49.4^***^

* *p < 0.05;*

** *p < 0.01;*

*** *p < 0.001*.

### Structural Equation Model

**Figure 1:**
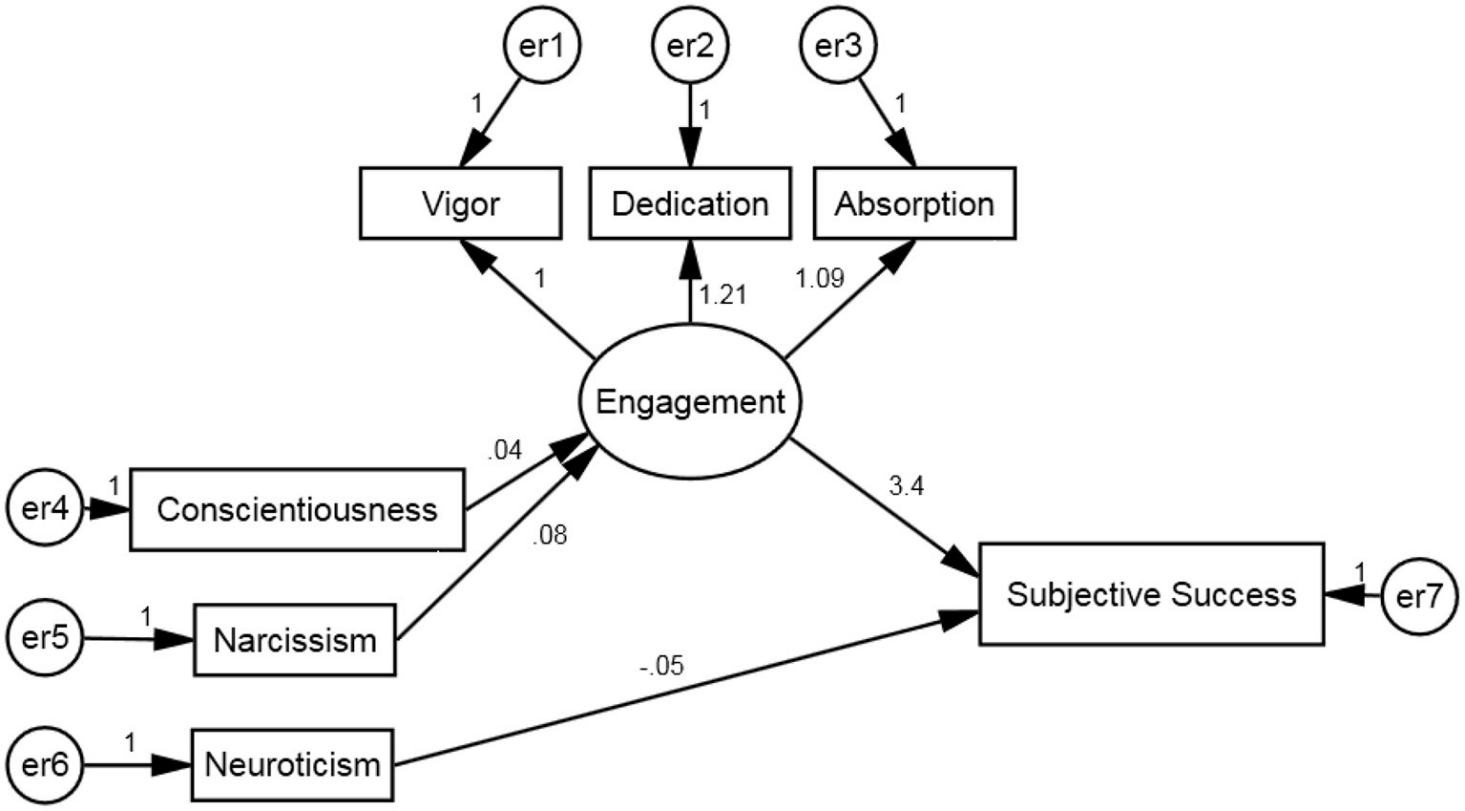
SEM predicting Subjective Success.

A structural equation model (SEM) was analyzed in order to assess the potential mediating effect of engagement on the relationship between personality and subjective success. The model was analyzed in R (version 3.3.0), using the Lavaan package (Rosseel, 2012; version 0.5-20). As data was not normally distributed, maximum likelihood with robust standard errors was used for parameter estimation. The tested model entered Subjective Success and the HPTI and Dark Triad personality traits as observed variables. Engagement was entered as a latent variable, made up of Vigor, Dedication, and Absorption. Non-significant variables were removed in a stepwise manner from the model until only significant predictors remained. See Figure 1 for details of SEM.

Whilst the model yielded a significant chi- square statistic (χ2(11) = 45.4, *p* < 0.001), researchers have indicated that large sample sizes artificially inflate chi-square values, causing a rejection of the model. However, analysis of other fit indices indicated initially that the model was a good fit for the data: CFI = 0.97; TLI = 0.95. However, RMSEA indicated a sub-par fit: RMSEA = 0.102 [Lower 90% CI = 0.075; Upper 90% CI = 0.135]. Recent papers have demonstrated how models with small degrees of freedom are more likely to have RMSEA values that indicate poor fit, partially due to the greater range in RMSEA confidence interval (Kenny et al., 2015). Assessing the combination of fit indices, the model appears to demonstrate adequate fit.

The final model indicated that Conscientiousness (ß = 0.04; *p* < 0.001) and Narcissism (ß = 0.08; *p* < 0.001) had positive direct effects on Engagement. Neuroticism was the only personality trait noted to have a direct significant impact on Subjective Success (*ß* = −0.05; *p* < 0.001), with Engagement also having a significant impact (*ß* = 3.4; *p* < 0.001).

The mediating role of Engagement was tested by measuring the indirect impact of conscientiousness and Narcissism would have on Subjective Success. Previous research has suggested utilizing bootstrapping procedures when measuring indirect effects (Shrout and Bolger, 2002; Cheung and Lau, 2007), so 1,000 bootstrap samples were created. Using the bias-corrected percentile method, significant indirect effects were noted for both Conscientiousness (*ß* = 0.12; *p* < 0.001) and Narcissism (*ß* = 0.26; *p* < 0.001). These results indicate that Engagement fully mediates the effect of Conscientiousness and Narcissism on subjective success.

## Discussion

The essential aim of this study was to explore three correlates of the six high potential traits identified by the HPTI namely Dark Triad, Work Engagement and Subjective Success. The correlational result showed nearly all of the hypotheses were confirmed the exceptions being H1 (age) and H9 (Competitive). In many ways these results replicated previous studies (Teodorescu et al., 2017). Four of the six high-flier traits were significantly correlated with success particularly Courage and Conscientiousness. Indeed, practically all studies on bright-side correlates of all measures of work success highlight the role of Conscientiousness which is be expected given that this trait indicates that a person is hard-working, organized, planful, and responsible (Teodorescu et al., 2017).

There was also consistent evidence in the correlational, regression and SEM analysis that low Adjustment/high Neuroticism was correlated with subjective success as indeed it is with objective success (Furnham, 2018). The explanation for this well-established finding is that Neuroticism is associated with anxiety, depression and most of all poor decision making (Sutin et al., 2009; Treglown et al., 2016). The current interest in trait Resilience is testimony to the observation that it is a fundamentally important feature of success at work, and something that those with low Adjustment lack (Judge et al., 1999).

It is interesting that Courage was the only significant high-flier variable in the regression, along with low Adjustment (Neuroticism). Later versions of the HPTI label Courageous as Risk Approach and note those with high scores handle risk and conflict well and do not put off holding difficult conversations, addressing underperformance, providing feedback or disagreeing profoundly with others. They are willing to take on risky and challenging projects: seeing risks are opportunities.

However, the most interesting results lay in the regression and the SEM. The first result of note showed that when the demographic, high-flier and dark-triad measures were regressed onto the three engagement facet scores as criteria variables, the clearly consistent result was that Narcissism was by far the biggest predictor accounting for around a quarter of the variance. This showed that those prone to inflated opinions about their abilities viewed themselves to be more work engaged, despite the fact that this is probably not the case (Furnham, 2018). Indeed, it could well be that the correlation between subjectively and objectively/observed measures of success are lowest in Narcissists as their abiding characteristic is distorting and inflating what ever success they achieve. On the other hand, their self-evidence self-confidence may work in their favor leading others to believe in their abilities and performance.

It is noteworthy that few of the other variables were systematically related to engagement. The correlational results showed that Curiosity, Conscientiousness, and Courage were consistently and significantly associated with all three Engagement factors. Interestingly, only one of the correlations with Adjustment was significant which was the same for Ambiguity Acceptance where the correlation was indeed negative. Possibly, these two traits are very sensitive to the particular nature of the job in the sense that in some jobs they are very engaged and in others not at all.

We found that neither our “bright-side” high flier, nor “dark-side” dark triad traits were strongly related to either job engagement or perceived success. What is most striking about the regressions shown in Tables 3–5 is the fact that only one of the nine high-flier, dark-side traits were significant predictors in two of the three analyses. In other words, relatively few of the traits were related to either engagement or subjective success.

Our results do not confirm those of Spurk et al. (2016) who used different measures of the independent variable (DT) and dependent variable (five item rating of career success) and on a different population. In that study they found little evidence of any relationship between subjectively rated success and the DT.

The results show best how the predictor variables (high-flier traits, dark-triad traits, job engagement) related to the criterion variable: subjectively rated job success. The regression showed that Adjusted males with high scores on trait Courage and Narcissism and who claimed high engagement on Vigor and Dedication believed they were more successful at work. One interesting question is whether indeed we find similar associations when we look at the data on personality correlates of “actual,” objectively defined work success? The data show traits Conscientiousness and Adjustment predict both Engagement and work success which is a well-established finding (Furnham, 2018).

What the SEM analysis shows is that Engagement is a moderator variable for Conscientiousness but not Adjustment. Presumably Conscientiousness is often expressed in work engagement, particularly Vigor defined as high energy, resilience, a willingness to invest effort on the job, the ability to not be easily fatigued, and persistence when confronted with difficulties; and dedication defined as a strong involvement in work, enthusiasm, and sense of pride and inspiration.

Finally, there is the all-important issue of national and corporate culture differences. It is quite possibly that some cultures define and value very different features of “success at work” which would mean it had different correlates. Clearly, this is worth exploring in future work.

Like all this study had limitations: this was a cross-sectional, self-report study. Thus, we cannot establish whether traits are in some sense causes as well as correlates of job success, though other studies would suggest the former. We only had a subjective measure of success and no details on individuals' job histories or actual record of success like salary or promotions. Indeed, some of the success at work measure referred to educational success which is not the same as work success although items were positively correlated and the alpha of the five items acceptable.

There is also the ever-present problems of social desirability which may exaggerate the engagement responses but repress the dark-side variables. This may have in-part accounted for the relationship between Narcissism, engagement, and success. We know that Narcissism has less negative outcomes than Psychopathy or Machiavellianism, also in the work context, but that they tend to overestimate their successes as we found in our data. Whilst the size of the sample was sufficient for statistical purposes there are questions about its representativeness of a range of working people.

Equally, our statistical methods could be open to criticism. Thus, it could be argued that the regressions are unjustified because of the high correlation between the different measures of engagement. Next, the fit in the SEM model would be too low for some statisticians and would suggest collecting more data and/or a different analysis.

Nearly all studies in work psychology have highlighted two traits as being most closely related to all work outcomes namely Conscientiousness and Neuroticism. This study was no exception which has clear implications in job selection (Furnham, 2018). It also demonstrated the role of trait Narcissism in self-rated success though it is unclear whether this is a manifestation of impression management or self-delusion. Certainly, the data suggest that people with high Narcissism scores can be problematic in the work-place because of their focus on themselves rather than their team or the organization as a whole (Furnham, 2021).

## Data Availability Statement

The raw data supporting the conclusions of this article will be made available by the authors, without undue reservation.

## Ethics Statement

The studies involving human participants were reviewed and approved by CEHP/514/2017. The patients/participants provided their written informed consent to participate in this study.

## Author Contributions

AF conceived of, and wrote the paper. LT did all the statistical analysis. Both authors contributed to the article and approved the submitted version.

## Conflict of Interest

LT was employed by company Thomas International. The remaining author declares that the research was conducted in the absence of any commercial or financial relationships that could be construed as a potential conflict of interest.
